# Differing spectrum of HIV-associated ophthalmic disease among patients starting antiretroviral therapy in India and South Africa

**DOI:** 10.1111/j.1365-3156.2010.02712.x

**Published:** 2011-03

**Authors:** Sophia Pathai, Clare Gilbert, Helen A Weiss, Matthew McNally, Stephen D Lawn

**Affiliations:** 1 Department of Clinical Research, London School of Hygiene and Tropical MedicineUK; 2 Medical Research Council Tropical Epidemiology Group, London School of Hygiene and Tropical MedicineUK; 3 Hannan Crusaid Treatment CentreGugulethu, Cape Town, South Africa; 4 Desmond Tutu HIV Centre, University of Cape TownSouth Africa

**Keywords:** opportunistic infections, cytomegalovirus, cytomegalovirus retinitis, ophthalmology, Africa, Asia

## Abstract

Differences in the prevalence and spectrum of HIV-associated ophthalmic disease in Africa and Asia are not well documented. We studied two comparable cohorts of patients initiating antiretroviral therapy in Mumbai, India, and Cape Town, South Africa. The prevalence of HIV-associated ophthalmic disease was higher in the Indian population (17.5%) than in the South African population (12.1%). This was largely because of vitreo-retinal opportunistic infections (11.4%*vs*. 2.6%, respectively), notably cytomegalovirus retinitis. This difference persisted after adjusting for confounding factors (adjusted odds ratio = 11.32, 95% confidence interval: 2.67–48.13), confirming a marked geographical difference in the prevalence of HIV-associated retinal disease.

HIV-associated eye disease is estimated to affect 50–75% of HIV-infected people worldwide at some point during their disease course (Kestelyn & Cunningham 2001). In resource-limited settings, patients typically start antiretroviral therapy (ART) at low CD4 counts and may be at high risk of ocular opportunistic infections (OIs). Retinal OIs such as cytomegalovirus retinitis (CMVR) can lead to chronic visual impairment or blindness if untreated. Additionally, immune reconstitution phenomena such as immune recovery uveitis after ART initiation may increase ocular morbidity (Karavellas *et al.* 1998; Price *et al.* 2001). Identification and treatment of ocular disease in HIV-infected individuals is therefore important.

Reports suggest that the prevalence and spectrum of HIV-related eye disease differs geographically. In Asia, the predominant manifestation is CMVR, followed by other retinal OIs. Estimates are highest in south-east Asia, where CMVR affects 27–33% of HIV-infected individuals (Biswas *et al.* 2000; Ausayakhun *et al.* 2003; Gharai *et al.* 2008; Shah *et al.* 2008; Pathai *et al.* 2009). In contrast, the reported prevalence of CMVR in sub-Saharan Africa is low (0–16.5%), whereas complications affecting the anterior segment of the eye such as herpes zoster and molluscum contagiosum are more frequently reported (Lewallen *et al.* 1994; Jaffar *et al.* 1999; Kestelyn 1999). However, it is difficult to draw firm conclusions about these differences as study populations have differed with regard to levels of immunodeficiency, ART exposure and methods of case ascertainment.

We previously reported on the prevalence of ophthalmic disease among patients enrolling in an ART service in Mumbai, India (Pathai *et al.* 2009), and have since implemented the same study protocol, using the same study personnel, in a comparable cohort of patients starting treatment in a well-characterised ART service in Cape Town, South Africa (Lawn *et al.* 2006, 2007). The aim of this paper was to directly compare the profile of HIV ocular manifestations in these two populations.

Data collection comprised demographic information, standardised ocular symptom screening and a full ophthalmic examination among patients starting ART as previously detailed (Pathai *et al.* 2009). Prevalence of HIV ocular disease was estimated, and factors associated with disease were compared between sites using the chi-squared test. Multiple logistic regression analysis was used to examine associations between retinal OIs and demographic/clinical characteristics. Three hundred and six patients were studied (157 in Cape Town and 149 in Mumbai). Patients from the African and Indian study sites had similar demographic and clinical characteristics although the Cape Town cohort had a lower median CD4 cell count (Table 1).

**Table 1:** Summary characteristics of the two cohorts of patients enrolling for antiretroviral therapy

Variable	Cape Town (*n* = 157)	Mumbai (*n* = 149)	*P*-value
Age (years) median ± SD	34 ± 10.1	36 ± 8.3	0.12
CD4 count (cells/μl) median (IQR)	143 (59–199)	180 (106–253)	0.01
History of past or current	80	79	0.72
TB	50.1% (42.8–59.0)	53.0% (44.7–61.2)	
*n*, % (95% CI)			
WHO clinical stage	99	83	0.19
3 or 4	63.1% (55.0–70.6)	55.7% (47.3–63.8)	
*n*, % (95% CI)			

SD, standard deviation; *n*, number; IQR, interquartile range; 95% CI, 95% confidence interval.

The prevalence of overall HIV-related eye disease was higher in the Mumbai (17.5%) than in the Cape Town cohort (12.1%; Table 2). The evidence for a difference was strongest among those with CD4 count <200 cells/μl and those with advanced WHO clinical stage. When stratifying by specific diagnoses, it was clear that the overall difference was largely because of the higher prevalence of retinal OIs in the Mumbai cohort, specifically CMVR (Table 2).

**Table 2:** Prevalence of HIV-related eye disease within different risk groups in Cape Town and Mumbai populations

	Overall study population			Participants with CD4 count <200 cells/μl			Participants staged at WHO clinical stage 3 or 4		
			
Prevalence % (95% confidence interval), *n*	Cape Town (*n* = 157)	Mumbai (*n* = 149)	*P*	Cape Town (*n* = 120)	Mumbai (*n* = 84)	*P*	Cape Town (*n* = 99)	Mumbai (*n* = 83)	*P*
Overall HIV ocular disease	12.1% (7.4–18.3)	17.5% (11.7–24.5)	0.19	13.3% (7.8–20.7)	23.8% (15.2–34.3)	0.05	15.5% (8.7–23.8)	27.2% (18.4–38.6)	0.05
	19	26		16	20		15	23	
Overall retinal opportunistic infections†	2.6% (0.7–6.4)	11.4% (6.8–17.6)	0.003	2.5% (0.5–7.1)	15.5% (8.5–25.0)	0.001	4.0% (1.1–10.0)	19.3% (11.4–29.4)	0.001
	4	17		3	13		4	16	
Cytomegalovirus retinitis (CMVR)	1.3% (0.1–4.5)	8.7% (4.7–14.4)	0.003	0.8% (0.02–4.5)	11.9% (5.8–20.8)	0.001	2.0% (0.2–7.1)	14.4% (7.7–23.9)	0.002
	2	13		1	10		2	12	
Ocular TB and toxoplasmosis	1.3% (0.1–4.5)	2.7% (0.7–6.7)	0.38	1.6% (0.2–5.9)	3.6% (0.7–10.0)	0.39	2.0% (0.2–7.1)	4.8% (1.3–11.8)	0.29
	2	4		2	3		2	4	
Other conditions									
Optic neuropathy	3.8% (1.4–8.1)	2.0% (0.4–5.8)	0.5	4.2% (1.4–9.5)	0 (0)	0.08	4.0% (1.0–10.0)	2.4% (0.3–8.4)	0.69
	6	3		5			4	2	
HIV retinopathy	5.1% (2.2–9.8)	4.7% (1.9–9.4)	0.87	5.0% (1.9–10.6)	8.3% (3.4–16.4)	0.34	5.1% (1.7–11.4)	6.0% 2.0–13.5	0.77
	8	7		6	7		5	5	

*NB – participants could have more than one condition, so values within subsets may not reflect overall total.

† Retinal opportunistic infections: CMVR, choroidal TB, toxoplasmosis.

The difference in the risk of retinal OI between sites persisted after adjusting for age, gender and other *a priori* confounders (CD4 count, WHO clinical stage, visual impairment and ocular symptoms), with an adjusted odds ratio = 11.32, 95% confidence interval (CI): 2.67–48.13 (Table 3). In addition, low CD4 cell counts and advanced WHO clinical stages of disease were also independently associated with the prevalence of retinal OIs (Table 1).

**Table 3:** Multivariable analysis to investigate relationship between HIV-associated vitreo-retinal opportunistic infections and demographic, clinical and ophthalmic factors

Variable	Multivariable model* Odds ratio (95% confidence interval)	*P*-value (Wald test)
Gender		
Male	1	0.57
Female	0.71 (0.22–2.35)	
Age group (years)		
16–30	1	0.5
31–35	1.12 (0.28–4.45)	
36–40	0.65 (0.15–2.78)	
41–45	0.14 (0.01–1.52)	
>46	0.83 (0.17–3.93)	
Geographical location		
Cape Town	1	0.001
Mumbai	11.32 (2.67–48.13)	
CD4 count (cells/ml)		
>200	1	0.02
51–200	1.39 (0.38–5.07)	
0–50	6.98 (1.93–31.72)	
WHO clinical stage		
1 or 2	1	0.007
3 or 4	17.39 (2.21–137.14)	
Visual impairment		
None	1	0.07
Present	3.80 (0.89–16.17)	
Ocular symptoms		
No	1	0.63
Yes	1.49 (0.29–7.57)	

* Adjusted for all other factors in model.

Our data confirm that the spectrum of HIV-related eye disease differs between these two study sites in India and Africa. The use of identical study protocols and study personnel, as well as demographically and clinically similar study populations, suggests that these findings can be reliably compared. The low prevalence of retinal OIs within the Cape Town cohort (2.6%) is consistent with other data from the region (Lewallen *et al.* 1994; Jaffar *et al.* 1999; Kestelyn 1999). Similarly, our prevalence estimate of CMVR in Mumbai is in agreement with other reports from Asia *(*Biswas *et al.* 2000; Ausayakhun *et al.* 2003; Gharai *et al.* 2008; Shah *et al.* 2008).

A previously proposed hypothesis for this difference is that patients with advanced immunodeficiency in the African region have high mortality and do not survive to develop CMVR and other complications (Jaffar *et al.* 1999). However, this would not explain our findings whereby the geographical location was a strong independent predictor of the prevalence of ophthalmic disease after adjustment for CD4 cell count and WHO stage of disease. Previous studies have shown that CMV seroprevalence is similar in the two populations (Kothari *et al.* 2002; Cannon *et al.* 2010; Chakraborty *et al.* 2010; Rabenau *et al.* 2010), suggesting lack of exposure to CMV within the Cape Town cohort is an unlikely reason for this difference. The disparities in prevalence are unclear but could potentially be related to genetic differences at the host or virus level (Jabs 1995; Jaffar *et al.* 1999). There are no reports to date of geographical differences in the epidemiology of non-ocular systemic CMV disease, possibly suggesting that host ophthalmic factors may also be involved. The prevalence of HIV retinopathy was similar in both groups, and these non-infectious retino-vascular pathological processes may occur independently of CMV-mediated mechanisms.

In conclusion, the higher prevalence of HIV-associated ophthalmic disease in the Indian study population was largely because of the higher prevalence of CMVR. Further studies are needed to document other geographical variations in ocular HIV epidemiology and investigate the underlying mechanisms. Most cases of CMVR in the Indian population were not detectable with symptom screening (Pathai *et al.* 2009), and this indicates the need for careful retinal screening in this patient population in those with low CD4 cell counts. Routine retinal screening by ophthalmologists may not be easily available within all settings, and alternative cost-effective strategies to detect retinal OIs within this population may need to be devised.
